# Diagnostic accuracy of diffusion-weighted imaging with conventional MR imaging for differentiating complex solid and cystic ovarian tumors at 1.5T

**DOI:** 10.1186/1477-7819-10-237

**Published:** 2012-11-09

**Authors:** Ping Zhang, Yanfen Cui, Wenhua Li, Gang Ren, Caiting Chu, Xiangru Wu

**Affiliations:** 1 Department of Obstetrics and Gynecology, Xinhua Hospital affiliated to Shanghai JiaoTong University School of Medicine, Shanghai, 200092, China; 2 Department of Radiology, Xinhua Hospital affiliated to Shanghai JiaoTong University School of Medicine, Shanghai, 200092, China; 3 Department of Pathology, Xinhua Hospital affiliated to Shanghai JiaoTong University School of Medicine, Shanghai, 200092, China

**Keywords:** Ovary, Ovarian tumors, Diffusion-weighted imaging, Apparent diffusion coefficients

## Abstract

**Background:**

Preoperative characterization of complex solid and cystic adnexal masses is crucial for informing patients about possible surgical strategies. Our study aims to determine the usefulness of apparent diffusion coefficients (ADC) for characterizing complex solid and cystic adnexal masses.

**Methods:**

One-hundred and 91 patients underwent diffusion-weighted (DW) magnetic resonance (MR) imaging of 202 ovarian masses. The mean ADC value of the solid components was measured and assessed for each ovarian mass. Differences in ADC between ovarian masses were tested using the Student’s *t*-test. The receiver operating characteristic (ROC) was used to assess the ability of ADC to differentiate between benign and malignant complex adnexal masses.

**Results:**

Eighty-five patients were premenopausal, and 106 were postmenopausal. Seventy-four of the 202 ovarian masses were benign and 128 were malignant. There was a significant difference between the mean ADC values of benign and malignant ovarian masses (p < 0.05). However, there were no significant differences in ADC values between fibrothecomas, Brenner tumors and malignant ovarian masses. The ROC analysis indicated that a cutoff ADC value of 1.20 x10^-3^ mm^2^/s may be the optimal one for differentiating between benign and malignant tumors.

**Conclusions:**

A high signal intensity within the solid component on T2WI was less frequently in benign than in malignant adnexal masses. The combination of DW imaging with ADC value measurements and T2-weighted signal characteristics of solid components is useful for differentiating between benign and malignant ovarian masses.

## Background

Ovarian tumors are the leading indication for gynecologic surgery, and the preoperative characterization of complex solid and cystic adnexal masses is crucial for informing patients about possible surgical strategies. Recently, in order to improve quality of life, laparoscopic surgery has been increasingly used for the treatment of ovarian tumors that are believed to be benign. For malignant ovarian lesions, there is no evidence that laparoscopy for the management of early stage ovarian tumors may improve the quality of life [1-3].

Ultrasonography (US) is the first-line imaging modality for adnexal lesions and is a particularly useful preoperative test for the characterization of noncomplex masses. Magnetic resonance imaging (MRI) may be of great help in identifying malignant lesions before surgery, particularly when US findings are suboptimal or indeterminate [4-10]. MRI can reveal morphologic characteristics such as papillary projections, nodularity, septa, solid portions and signal intensity on T1- and T2-weighted images, but none of these criteria reliably distinguish between benign and malignant tumors. The use of magnetic resonance (MR) diffusion-weighted imaging (DWI) may improve MR characterization of ovarian lesions. Diffusion-weighted imaging is sensitive to changes in the microdiffusion of water into both intracellular and extracellular spaces. Differences in the apparent diffusion coefficient (ADC) of benign and malignant complex adnexal masses have been reported [11-13]. Kayayama *et al*. [11] and Fujii *et al*. [14] assessed the feasibility of DWI for the differentiation of benign and malignant ovarian lesions. They concluded that the ADC values of cystic and solid components were not useful for differentiating between lesions. However, their data included endometrial cysts, mature cystic teratomas, and fibromas and fibrothecomas, in which hemorrhagic contents, sebaceous materials and fibrous tissue may cause an increase or a reduction in signal on DWI.

The purpose of this study was to clarify the relationship between the ADC values of the solid components of benign and malignant ovarian lesions, and to evaluate the supplementary use of ADC values of the solid components for differentiating between benign and malignant lesions.

## Methods

### Patients

Our institutional review board approved the study and waived the requirement to obtain written informed consent. A total of 601 female patients evaluated for ovarian tumors between January 2005 and December 2011 were enrolled. A retrospective review of MRI data was undertaken. The MRI studies met the following inclusion criteria for the study: (1) MRI was performed using a 1.5T magnet, and (2) both conventional MRI with DWI and dynamic contrasted-enhancement MRI were performed. In addition, the diagnosis was confirmed by surgery and pathological examination. Of the 601 patients, 383 were excluded due to the presence of endometriomas (n = 73), mature cystic or immature teratomas (n = 39), and purely cystic ovarian lesions (including simple cysts and purely cystic adenomas) with thin and regular walls and/or septa (n = 232). A further 27 were excluded due to failed fat suppression or artifacts. The remaining 191 patients with 202 complex adnexal masses were included in the study.

### MR protocol

All subjects underwent MRI with a 1.5T MR unit (Twinspeed, GE Medical Systems, Milwaukee, WI, USA). The imaging protocol involved axial non-contrast T1-weighted (TR/TE, 400-600/10-14 ms) and axial T2-weighted (TR/TE, 4,000-6,000/100-120 ms) imaging performed with a chemical shift-selective fat saturation pulse using the following parameters: slice thickness, 5 mm; gap, 1 mm; field of view (FOV), 32 to 42 cm; matrix, 256 **×** 256; and excitation, 2. Sagittal T1-weighted and T2-weighted (TR/TE, 3,000-6,000/100-110 ms) fast spin-echo imaging without chemical shift-selective fat saturation pulse was also performed, as well as post-contrast enhanced axial and sagittal T1-weighted imaging using parameters as described above, apart from a slice thickness of 5 mm. Diffusion-weighted MRI was acquired in the axial plane prior to administration of contrast medium using a single-shot echo-planar imaging sequence (TR/TE effective range, 8,000-10,000/70-100; slice thickness/intersection gap, 5/1 mm; FOV, 32 to 42 cm; matrix, 128 × 128; excitation, 2. A b-value of 0 and of 1,000 s/mm^2^ was also applied in three orthogonal (Z, Y, and X) directions.

### MRI analysis

Conventional MRI and DWI imaging data were analyzed on an Advantage Windows workstation 4.2 (GE Healthcare, Milwaukee, WI, USA) by two radiologists (with 15- and 10- year experience in pelvic MRI, respectively), in consensus, having carefully reviewed all images. The solid component, according to a previously established classification by Timmerman *et al*. [7] included thickened septa, vegetation (papillary projection) and solid portions that showed enhancement post-injection. The cystic component was defined as tissue with homogeneous long T1 and T2 characteristics or different signal intensities on T1- or T2-weighted MR images, and showed no enhancement post-injection. The signal intensity of solid components on T2-weighted MR images was defined as low or intermediate (equal to or greater) compared to that of the outer myometrium. The signal intensity of the cystic and solid components on DWI at b = 1,000 s/mm^2^ was classified as intermediate or low compared to that of serous fluid (urine or cerebrospinal fluid).

### Data calculation and analysis

The solid components of the lesions were identified on T2-weighted and post-contrast T1-weighted images, and were matched on ADC maps. The ADC values of the solid components of each tumor were measured on DW images by a radiologist using an Advantage Windows workstation 4.2 (GE Healthcare), using the manufacturer’s software (FuncTool; GE, Medical Systems, Milwaukee, WI, USA). In order to minimize variability, the largest possible regions of interest (ROIs), which varied from 15 to 150 mm^2^, were manually placed in the solid parts of the tumor. When the lesion exhibited irregular or heterogeneous solid components, numerous vegetations or thickened irregular septa, between two and five ROIs were drawn within the targeted components and the mean ADC value was used in the analysis.

### Statistical analysis

Surgical pathological findings served as the reference standard for assessment of ovarian tumors. All analyses were performed using SPSS version 13.0 for Windows (SPSS, Chicago, IL, USA). Differences in mean tumor ADC values between benign and malignant groups were evaluated using Student’s *t*-test. A *P*-value < 0.05 was considered statistically significant. Receiver operating characteristic (ROC) curve analysis was performed in order to assess the diagnostic performance of the mean ADC values in terms of characterization of benign and malignant ovarian tumors.

## Results

### Clinical demographics

The surgical pathological types of 202 complex adnexal masses in 191 patients can be seen in Table 1. The mean patient age was 56.52 ±15.31 (mean±SD; range, 12 to 85) years. Eighty-five women (44.5%) were premenopausal and 106 (55.5%) were postmenopausal. Seventy-four (36.6%) of the 202 ovarian masses were benign, and 128 (63.4%) were malignant.

**Table 1:** Histological types and apparent diffusion coefficient (ADC) values of 202 ovarian masses

**Type of ovarian mass**	**Lesions, number**	**High SI on T2-WI**	**High SI on DWI**	**Range of ADC values**	**Mean ADC values**
Benign ovarian lesions	74	39 (52.7)	53 (71.6)	0.12, 1.81	1.22 ± 0.46
Serous cystadenoma	28	22 (78.6)	28 (100)	1.23, 1.81	1.52 ± 0.19
Mucinous cystadenoma	17	13 (76.5)	17 (100)	1.12, 1.79	1.48 ± 0.20
Fibrothecoma	18	3 (16.7)	3 (16.7)	0.40, 1.58	0.82 ± 0.44
Cystadenofibroma	6	0 (0)	0 (0)	0.12, 0.94	0.52 ± 0.26
Brenner tumor	5	1 (20)	5 (100)	0.88, 1.44	1.06 ± 0.23
Malignant ovarian lesions	128	108 (84.4)	128 (100)	0.56, 1.45	0.91 ± 0.20
Serous cystadenocarcinoma	35	26 (74.3)	35 (100)	0.66, 1.35	0.97 ± 0.20
Mucinous cystadenocarcinoma	27	20 (74.1)	20 (100)	0.65, 1.31	0.89 ± 0.19
Serous borderline tumor	12	10 (83.3)	10 (100)	0.78, 1.45	1.05 ± 0.19
Mucinous borderline tumor	9	8 (88.9)	9 (100)	0.75, 1.36	0.99 ± 0.17
Clear cell adenocarcinoma	8	8 (100)	8 (100)	0.69, 1.01	0.82 ± 0.13
Endometrioid adenocarconoma	6	6 (100)	6 (100)	0.71, 1.06	0.93 ± 0.11
Granulosa cell tumor	7	6 (85.7)	6 (100)	0.79, 1.33	0.98 ± 0.18
Undifferentiated adenocarcinoma	5	5 (100)	5 (100)	0.64, 1.19	0.83 ± 0.23
Metastatic tumors	19	19 (100)	19 (100)	0.56, 1.09	0.86 ± 0.15

Numbers in parentheses are percentages; ADC values, (mean ± SD × 10^-3^mm^2^/s); T2-WI, T2-weighted image; SI, signal intensity; DWI, diffusion-weighted imaging.

### MRI findings

**Figure 1:**
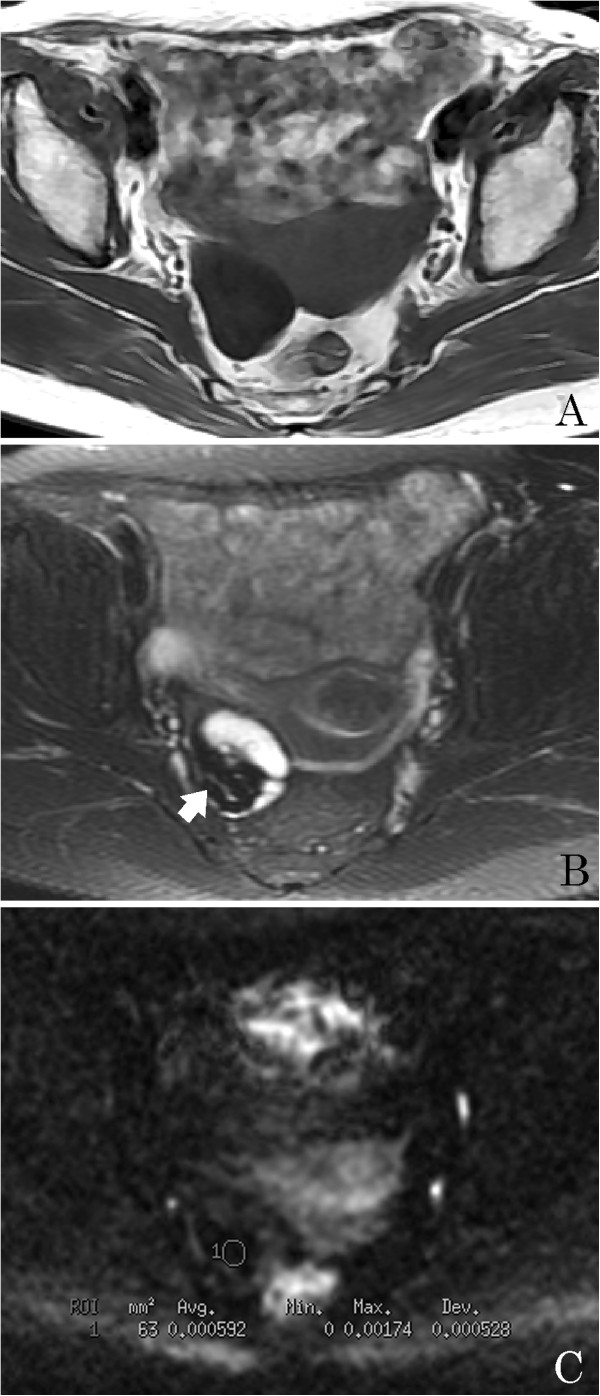
**A 67-year-old woman with an ovarian cystadenofibroma.** (**A**) Axial T1-weighted image shows a right ovarian mass with a hypointensity. (**B**) Axial T2-weighted image reveals that the solid part of the mass is hypointense (arrow) and the cystic component is hyperintense. (**C**) Axial diffusion-weighted imaging (DWI) reveals that the solid part of the mass has a very low signal intensity (circle 1, apparent diffusion coefficient (ADC) = 0.592 × 10^-3^ mm^2^/s).

**Figure 2:**
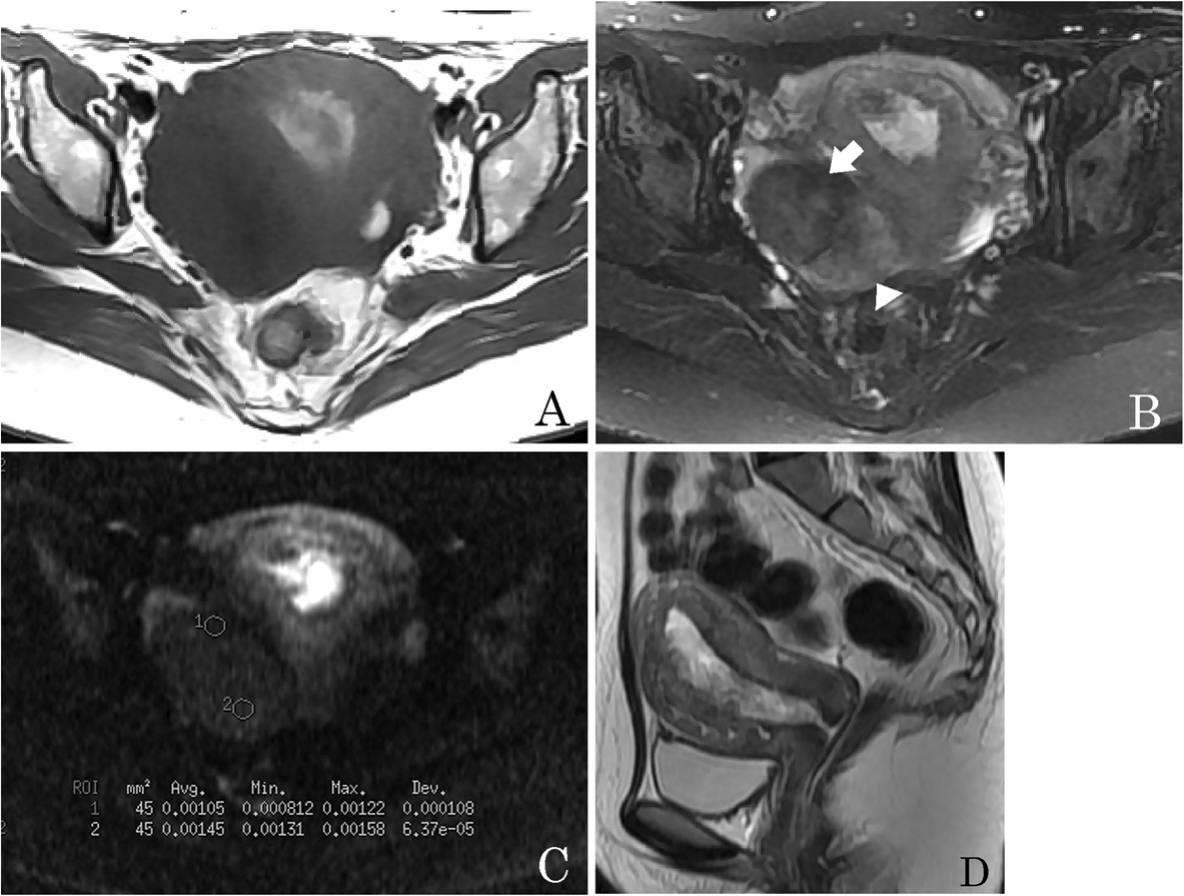
**A 27-year-old woman with an ovarian fibrothecoma.** (**A**) An axial T1-weighted image shows a right ovarian mass with hypointensity. (**B**) Axial T2-weighted image shows a solid heterogeneous signal mass; the area containing abundant fibrous tissue has marked low signal intensity (arrow) and the area containing an abundant thecoma component has intermediate signal intensity (arrowhead). (**C**) On axial diffusion-weighted imaging (DWI), the apparent diffusion coefficient (ADC) value of the fibrous tissue area is very low (circle 1, ADC = 1.05 × 10^-3^ mm^2^/s) and that of the thecoma component is very high (circle 2, ADC = 1.45 × 10^- 3^ mm^2^/s). (**D**) Sagittal T2-weighted image demonstrates endometrial thickening.

**Figure 3:**
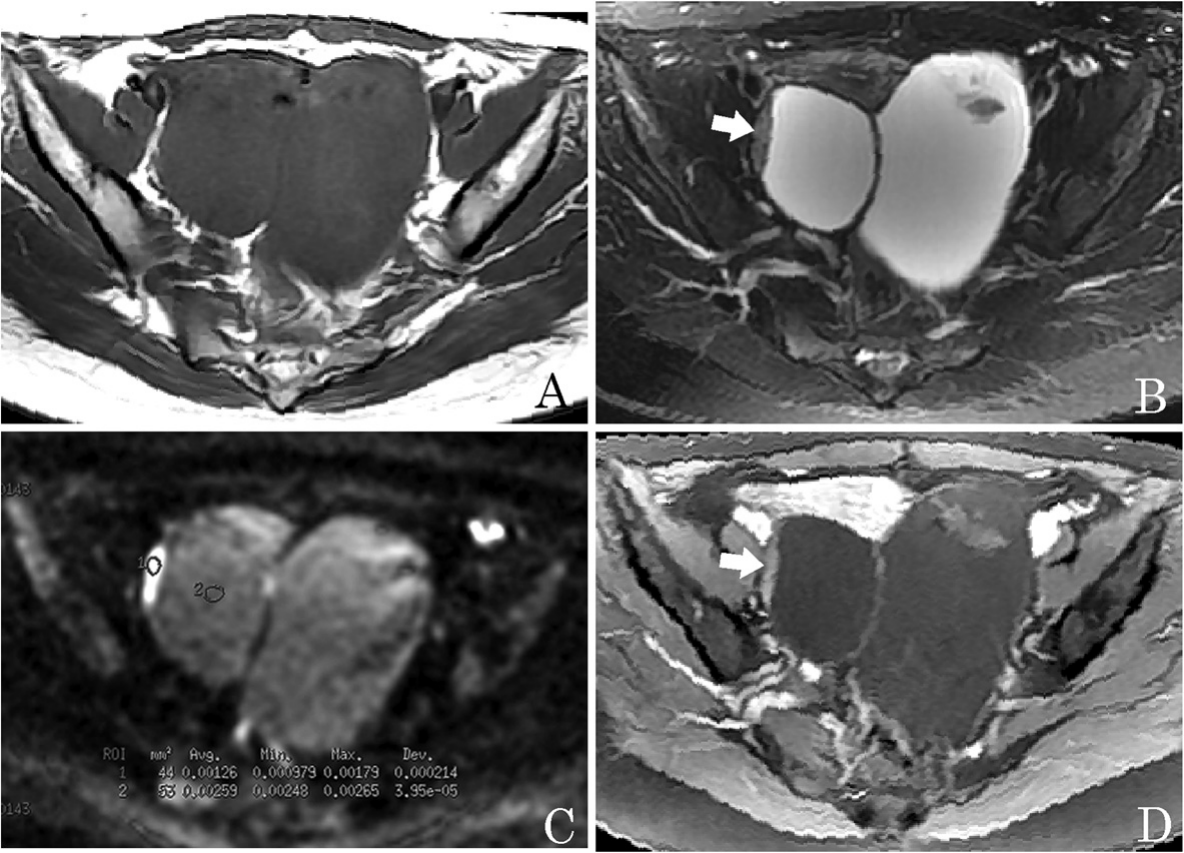
**A 28-year-young woman with bilateral benign serous ovarian cystadenomas.** (**A**) An axial T1-weighted image shows a pelvic tumor with hypointensity. (**B**) Axial T2-weighted image shows a complex adnexal tumor with solid and cystic components, and solid components as vegetations with intermediate signal intensity (arrow). (**C**) Axial diffusion-weighted imaging (DWI) obtained at b =1,000 s/mm^2^ shows the solid component as hyperintense with a high apparent diffusion coefficient (ADC) value (circle 1, ADC = 1.26 × 10^-3^ mm^2^/s), and the cystic component with an even higher value (circle 2, ADC = 2.59 × 10^- 3^ mm^2^/s). (**D**) Axial contrast-enhanced T1-weighted image demonstrates slight enhancement of the solid component (arrow).

**Figure 4:**
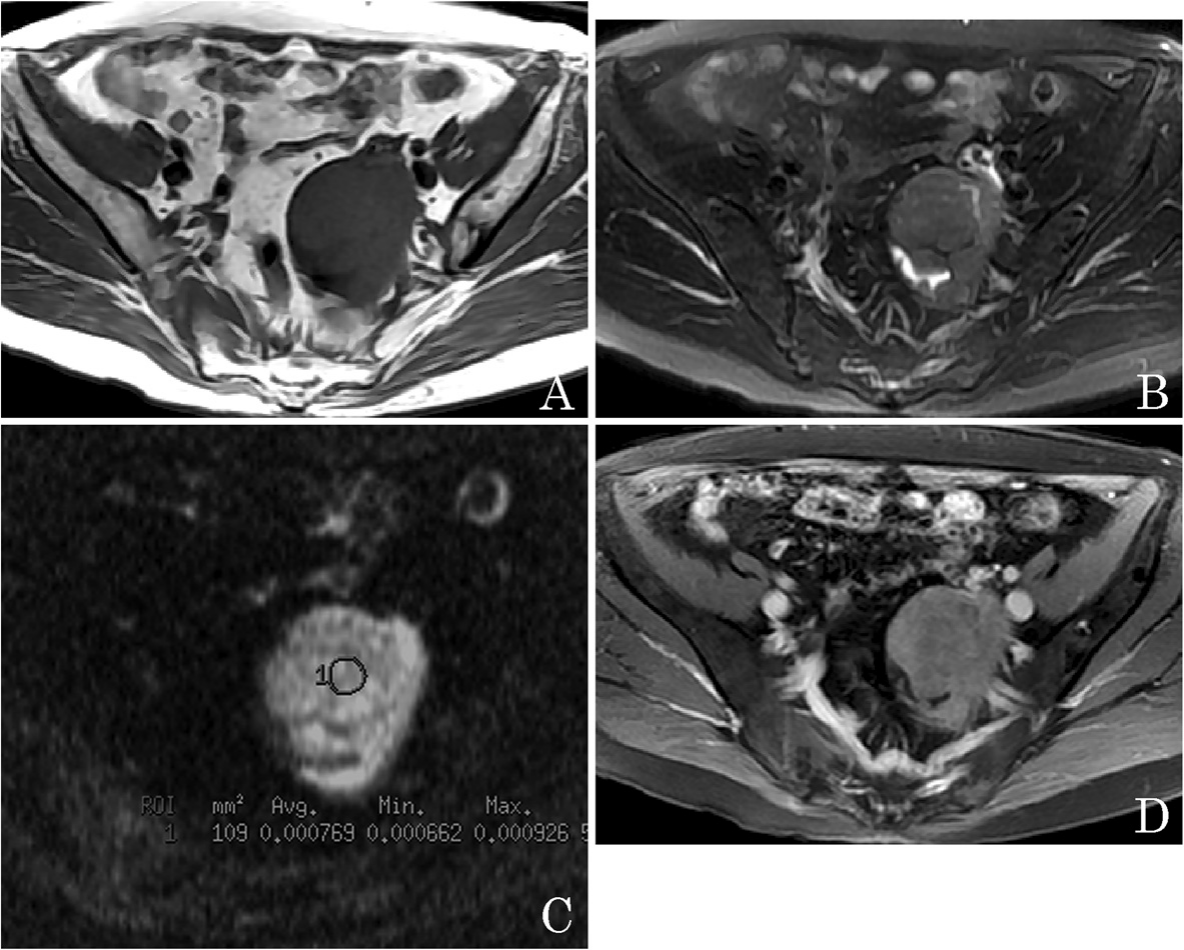
**A 63-year-old woman with a left ovarian cystadenocarcinoma.** (**A**) An axial T1-weighted image shows a left pelvic mass with isointensity. (**B**) Axial T2-weighted image reveals that the mass is a solid tumor with low signal intensity. (**C**) Axial diffusion-weighted imaging (DWI) obtained at b = 1,000 s/mm^2^ reveals the solid tumor as hyperintense with a lower apparent diffusion coefficient (ADC) value (circle 1, ADC = 0.769 × 10^-3^ mm^2^/s). (**D**) Axial contrast-enhanced T1-weighted image demonstrates marked enhancement of the solid part.

A high signal intensity within the solid component on T2-weighted MR images was observed less frequently in benign (39/74) than in malignant (108/128) adnexal masses (*P <* 0.01). The solid portion of 74 benign complex adnexal lesions contained homogeneous or heterogeneous low signal intensity on T2-weighted images in 100% of cystadenofibromas (Figure 1), 83.3% of fibrothecomas (Figure 2), 80.0% of Brenner tumors, 23.5% of mucinous cystadenomas, and 21.4% of serous cystadenomas (Figure 3). Among 128 malignant complex adnexal masses, only 20 (15.6%) displayed a low signal intensity of their solid components on T2-weighted images (Figure 4). On DWI, a high signal intensity at b = 1,000 s/mm^2^ within the solid component was observed less frequently in benign (71.6%) than in malignant (100%) adnexal masses (*P <* 0.05). Our results demonstrate that the following factors can be considered predictive of malignancy: the presence of a solid component with high or low signal intensity on T2-weighted images and high signal intensity on DWI with low ADC values (less than 1.20 × 10^-3^ mm^2^/s) at b = 1,000 s/mm^2^. On the other hand, the following factors can be considered predictive of a benign mass: the presence of a solid component with high or low signal intensity on T2-weighted images and high signal intensity on DW images with high ADC values (greater than 1.20 × 10^-3^ mm^2^/s), or low signal intensity on T2-weighted images and DW images with lower ADC values at b = 1,000 s/mm^2^.

### ADC analysis

**Figure 5:**
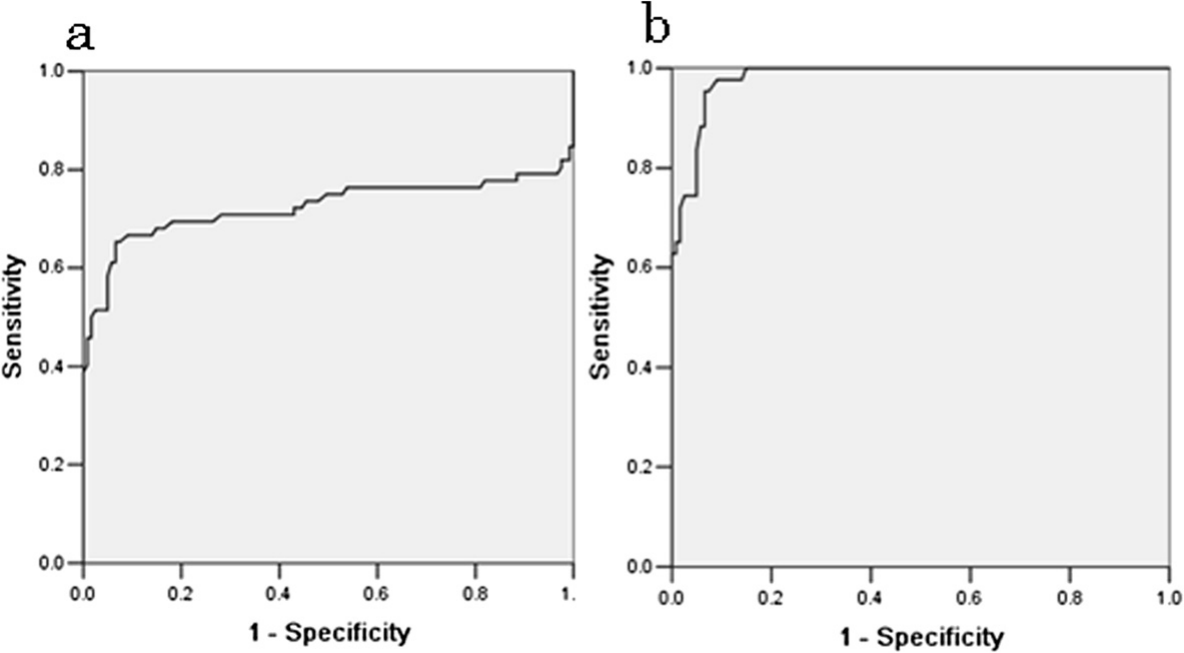
**Comparative diagnostic values of quantitative apparent diffusion coefficient (ADC) value parameters for discriminating between benign and malignant ovarian tumors.** When an absolute ADC value less than 1.20 × 10^-3^ mm^2^/s is predictive of malignancy, on receiver operating characteristic (ROC) analysis, the area under the curve (AUC) (**a**) is small (0.72), and when fibrothecomas, cystadenofibromas and Brenner tumors are excluded, the ROC AUC is the greatest (0.96), and thus it is probably the most effective factor for discriminating between benign and malignant ovarian masses (**b**).

The mean ADC values of the tumor solid components were determined for each group. There was much overlap between the range of values observed for benign and for malignant tumors. However the mean ADC value for benign tumors was 1.22 ± 0.46 × 10^-3^ mm^2^/s, and for malignant tumors 0.91 ± 0.20 × 10^-3^ mm^2^/s, which was a significant difference (*P <* 0.05). This result suggests that an ADC value ≥ 1.20 × 10^-3^ mm^2^/s may be the optimal cutoff for differentiating between benign and malignant tumors, with a sensitivity of 66.7%, a specificity of 90.9%, a positive predictive value (PPV) of 81.4%, a negative predictive value (NPV) of 82.1%, and an area under the curve (AUC) of 0.72. In our study, the mean ADC values were much lower for cystadenofibromas, fibrothecomas and Brenner tumors. There was no statistically significant difference between values for fibrothecomas, Brenner tumors and malignant tumors (*P* > 0.05), but there was a significant difference between values observed for cystadenofibromas and for malignant tumors (*P* = 0.002). When cystadenofibromas, fibrothecomas and Brenner tumors were excluded from the analysis, the sensitivity, specificity, PPV, NPV, and AUC for the optimal cutoff ADC value of 1.20 × 10^-3^ mm^2^/s for differentiating between benign and malignant tumors, were 97.7%, 90.1%, 86.6%, 99.1%, and 0.96, respectively (Figure 5).

## Discussion

Our findings demonstrate that the presence of high signal intensity in solid components of ovarian lesions on DW and T2-weighted imaging combined with low ADC values can be used to distinguish malignant from benign ovarian lesions. The results also suggest a potential role for DW imaging with quantitative analysis of ADC values in improving the diagnostic performance of ovarian MRI and yielding functional measures of the tumor microenvironment.

In the present study, ADC values are largely proportional to the ratio of extracellular and intracellular components, cell density, intracellular organelles, matrix fibers, and soluble macromolecules. Although some overlap in ADC values was observed between the benign and malignant groups, the mean ADC value of the 128 malignant ovarian tumors was significantly lower than that of the 74 benign ovarian tumors (*P <* 0.05). Our results suggest that an ADC value ≥ 1.20 × 10^-3^ mm^2^/s may be the optimal cutoff for differentiating between benign and malignant tumors. Furthermore, a sensitivity of 66.7%, a specificity of 90.9%, a PPV 81.4%, an NPV of 82.1%, and an AUC of 0.72, was observed with this ADC cutoff value. This result is consistent with previous reports [10,12,15,16]. In a study by Fujii *et al*. [14], the authors evaluated the contribution of DWI in combination with quantitative ADC analysis to the characterization of 123 ovarian lesions, which included 42 malignant and 81 benign lesions (including 7 fibromas, 18 mature cystic teratomas and 24 endometriomas). The results suggest that DW imaging of ovarian lesions and ADC values of the solid component are not useful for differentiating between benign and malignant ovarian lesions. This apparent discrepancy is probably due to the pathologic architectures of benign tumors. In our series, 83.3% (15/18) of fibrothecomas, 100% (6/6) of cystadenofibromas and 80% (4/5) of Brenner tumors demonstrated low signal intensity in the solid components on DWI and low ADC values, due to the presence of abundant collagen-producing fibroblastic cells and a dense network of collagen fibers within the extracellular matrix, which can restrict the Brownian motion of water molecules. Previous studies have also reported that endometriomas and mature teratomas exhibit low ADC values [11]. This could explain the observed absence of a difference between the mean ADC values of benign and malignant ovarian lesions in the study by Fujii *et al*. [14].

Furthermore, our study confirms that high signal intensity within solid components with low ADC values on DWI is a useful criterion for predicting malignancy. In our study, on T2-weighted images solid components of malignant tumors had high signal intensity in 84.4% and low signal intensity in 15.6%. On the other hand, 100% of solid components in malignant lesions had high signal intensity on DW images with low ADC values. This finding, that of low signal intensity on T2-weighted images and high signal intensity on DWI in solid components, may result from a reduction in both the extracellular matrix and the diffusion space of water protons in the extracellular and intracellular dimensions due to an increased nuclear to cytoplasmic ratio and hypercellularity. However, the low signal intensity on T2-weighted images and DWI in benign ovarian tumors such as fibrothecomas, cystadenofibromas and Brenner tumors, may be due to the high density of fibers, the low cellularity, and the low water content in both extracellular and intracellular spaces [13-20]. Our study suggests that low signal intensity on T2-weighted images and DWI with low ADC values within a solid component is an effective criterion for predicting the presence of benign disease.

There were several limitations to our study. First, the exclusion of endometriomas and mature cystic teratomas and pure cystic adenomas may have induced a selection bias. As recommended by Takeuchi *et al*. [16], the DWI findings of endometriomas and mature cystic teratomas are problematic, and most cases can be accurately diagnosed on conventional MRI. Secondly, drawing the ROI on DW images while viewing T2-weighted or contrasted T-weighted images may have resulted in an information bias, as DWI usually has poor spatial resolution. A better approach would be to fuse DW images at b = 1,000 × 10^-3^ mm^2^/s onto structural images in order to accurately position the ROI of solid components that show an abnormal signal on DW images. Thirdly, the reported ADC threshold value needs to be validated in a larger group of patients, as many factors can affect the ADC value, including magnetic susceptibility, spatial resolution, signal to noise ratio, and the pathophysiological characteristics of the ovarian lesion.

## Conclusion

DWI appears to be a useful method for differentiating between benign epithelial ovarian tumors with solid components and malignant ovarian tumors, and is associated with high sensitivity and specificity. The solid component within the complex adnexal mass that exhibits low signal intensity on T2-weighted images and DW images is invariably benign. In addition, a notable advantage of DWI is that it avoids further impairment of renal function, as the use of contrast medium is not necessary. For patients with renal failure, this would prevent the development of fibrosis.

## Competing interests

This work had no specific funding. The authors declare that they have no conflict of interest.

## Authors’ contributions

WH Li planned, designed and analyzed the study, collected data and wrote the manuscript, YF Cui, G Ren collected data, P Zhang designed the study, XR Wu contributed as a pathologist and helped in writing the manuscript. All authors read and approved the final manuscript**.**
